# Effects of antifoam agents on *Spodoptera frugiperda* 9 cell growth and baculovirus infection dynamics

**DOI:** 10.1186/s13036-025-00516-w

**Published:** 2025-05-10

**Authors:** Kristina Worch, Merlin Krause, Antje Burse

**Affiliations:** Department of Medical Engineering and Biotechnology, Ernst-Abbe-Hochschule, University of Applied Sciences, Carl-Zeiss-Promenade 2, 07745 Jena, Germany

**Keywords:** Cell culture, Insect cells, Sf9, Antifoam agent, Baculovirus

## Abstract

The baculovirus expression system is widely used for recombinant protein production. However, its scalability under shaking or stirring cultivation conditions remains a challenge due to foam formation which can negatively affect cell metabolism and viability, complicate process control, and ultimately lower productivity. Compared to other protein expression platforms, the effect of antifoam agents on insect cell culture has been rarely investigated. This study examines the influence of three antifoam agents—Antifoam 204 (AF204), polypropylene glycol (PPG), and a silicone-based compound (SAG471)—on *Spodoptera frugiperda* (Sf) 9 cell growth, viability, baculovirus infectivity, and infection dynamics. Dose-response experiments in adherent culture showed that high antifoam concentrations inhibited metabolic activity. In suspension culture, low concentrations of AF204 and PPG enhanced cell growth by reducing lag phase and population doubling time, while growth with SAG471 remained comparable to a no-antifoam control. In virus titer experiments, no effects on the plaque-forming ability of baculovirus particles could be observed. However, infection dynamics monitored in suspension cultures improved in the presence of all three antifoam agents, as shown by cell size increase, living cell stagnation, and enhanced single-cell fluorescence. Foam reduction experiments demonstrated that only SAG471 contributed to foam removal within a non-toxic concentration range. The results indicate that antifoam agents, depending on their concentration and composition, can enhance Sf9 cell growth and viability while potentially modulating cell membrane properties that could improve viral infection efficiency and transfection efficiency of exogenous material. This highlights the potential of antifoam agents for optimizing other virus-based expression systems in higher eukaryotic cells.

**Supplementary Information:**

The online version contains supplementary material available at 10.1186/s13036-025-00516-w.

## Introduction

Since the first mention of the baculovirus expression vector system in 1983, it has become one of the most important platforms for recombinant protein production, particularly for complex and post-translationally modified proteins—key components in vaccine development, drug production and structural biology [1–4]. Scaling up such platforms can be challenging due to foam formation, which can negatively impact cell metabolism and viability, affect protein structure, compromise sterility, hamper process control, and reduce overall production yields [5, 6]. Foaming can be mitigated through mechanical methods or incorporating antifoam agents which lower surface tension [5]. These agents are available in a variety of formulations, including polypropylene glycol-based, silicone-based and synthetic mixtures.

In prokaryotic systems, antifoam agents have been shown to enhance cell growth and production efficiency within certain concentration ranges [5, 7, 8]. However, in some cases these agents inhibit cell growth and product formation [7, 8]. For example, in *Saccharomyces cerevisiae*, industrial antifoam agents trigger stress responses that reduce fermentation performance [9], whereas in shake flask cultures of *Pichia pastoris*, their use has been associated with increased product yields [10]. In Chinese hamster ovary (CHO) cells, antibody production remained unaffected by the use of some antifoam agents, while others completely inhibited growth [11]. These effects are believed to result from interactions with the cell membrane [12], though the exact molecular mechanisms remain unclear.

Compared to other expression platforms, few studies have investigated the use of antifoam agents in baculovirus expression systems [13, 14]. Although insect cells are often cultivated in media containing Pluronic F-68 (e.g., Insect-XPRESS™ by Lonza, Sf-900™ II/III SFM, and Express Five™ SFM by Thermo Fisher Scientific), which reduces shear stress and foam formation [15, 16], foaming remains a major challenge—particularly in high-cell-density fermentation for industrial production, where additional aeration and stirring is needed. Thus, a deeper understanding of antifoam effects on insect cell cultivation is essential.

In this study, we selected three representative antifoam agents that are commonly used in fermentation processes but previously untested in insect cell cultivation. We investigated the effects of Antifoam 204 (AF204; organic silicone-free dispersion), polypropylene glycol (PPG), and a silicone antifoam compound (SAG471; silicone emulsion) on the growth and viability of *Spodoptera frugiperda* (Sf) 9 cells. Additionally, we examined the infectivity of recombinant *Autographa californica* multiple nucleopolyhedrovirus (AcMNPV) in the presence of these three antifoams. Our aim was to gain initial insight into their effects, identify optimal concentrations, and evaluate any beneficial or adverse impacts on a laboratory scale.

## Methods

### Cell culture

Sf9 cells (Leibniz Institute DSMZ, Braunschweig, Germany) were maintained in suspension in Insect-XPRESS™ medium (Lonza, Cologne, Germany). Cells were seeded at a density of 1.0 × 10^6^ cells/ml and passaged at densities above 7.0 × 10^6^ cells/ml. Unless otherwise stated, cultivation was performed in 50 ml culture vessels covered with ROTILABO^®^ cellulose culture plugs (size 13; Carl Roth, Karlsruhe, Germany) and aluminum foil. Cultivation took place in a MaxQ™ 6000 shaker (Thermo Fisher Scientific, Schwerte, Germany) at 27 °C and 180 rpm. Cell number and viability (trypan blue exclusion) were determined using a Countess 3 Automated Cell Counter (Thermo Fisher Scientific, Schwerte, Germany). Silanized glass vessels were used for suspension cultures of Sf9 cells [17].

### Evaluation of antifoam effects on cell culture and virus infectivity

AF204 (Merck, Darmstadt, Germany), PPG (Dow Olefinverbund GmbH, Schkopau, Germany), and SAG471 (Momentive Performance Materials GmbH, Leverkusen, Germany) were used in all experiments. Stock solutions of 2% (v/v) were prepared in distilled water and sterilized by autoclaving. Subsequent dilutions were made in medium, with no-antifoam controls containing medium only.

#### Viability assays

The toxic range of the antifoam agents was assessed using the 3-(4,5-dimethylthiazol-2-yl)2,5-diphenyltetrazolium bromide (MTT) reduction assay [18, 19]. Cell suspensions at different concentrations were seeded into 96-well cell culture plates (100 µl/well) and incubated at 27 °C for 4 h. Then, 11 µl antifoam solution was added. Based on prior experience with AF204 in *Escherichia coli* fermentation, a concentration range of 0.0001–0.02% was selected (K. Willing, personal communication, April 2024). Samples were run in duplicate or triplicate, and incubation was continued. After 24 h, 10 µl MTT (5 mg/ml in 1x PBS) was added to each well. Once formazan crystals became visible (approximately after 1 h), 100 µl lysis buffer (10% (w/v) SDS, 0.02 M HCl) was added and incubated for 30 min. Finally, absorbance at 570 nm (reference: 690 nm) was measured using a Spark^®^ Multimode Microplate Reader (Tecan Trading AG, Männedorf, Switzerland).

#### Cell growth assays

To each 10 ml cell suspension (0.5 × 10^6^ cells/ml), 100 µl of 0.01%, 0.05% or 0.1% antifoam solution was added to obtain final concentrations of 0.0001%, 0.0005% or 0.001%, respectively. Cell number and viability were measured over 9 days of cultivation. Samples were run in duplicate.

#### Virus titration assays

Plaque assays were performed to determine the titer of a stock solution of recombinant baculovirus (carrying the *mCherry* sequence [20] under the control of the polyhedrin promoter; created using the Bac-to-Bac^®^ Baculovirus Expression System [21]). Assays followed the Guide to Baculovirus Expression Vector Systems (BEVS) and Insect Cell Culture Techniques [22] using Insect-XPRESS™ medium and 12-well cell culture plates (0.25 × 10^6^ cells/well). Three virus stock aliquots were each supplemented with an antifoam agent (final concentration: 0.0001%) and incubated for 30 min at 180 rpm to allow interaction between the agents and the virus particles. Virus dilutions were then prepared (10^− 3^ (positive control), 10^− 5^, 10^− 6^, 10^− 7^, 10^− 8^ and a negative control with medium only; 800 µl/well; 2 wells per condition) and added to Sf9 cells for 1.5 h. Cultures were incubated at 27 °C in the dark. After hardening of the low-melting agarose layer (Carl Roth, Karlsruhe, Germany), by letting it dry under the working bench for 20 min, plates were sealed with parafilm. Plaques were visualized and counted after 7 days using neutral red staining (0.5 ml of 0.1 mg/ml working solution/well).

#### Infection assays

A 10 ml cell suspension (1.0 × 10^6^ cells/ml) containing antifoam (final concentration: 0.0001%) was cultured for 24 h. The cell number was determined, and the culture was infected with a virus stock volume sufficient to obtain a multiplicity of infection (MOI) of 1, based on titers calculated for the no-antifoam control. Infection was monitored every 12 h for 3 days by measuring cell size and fluorescence using a Countess 3 Automated Cell Counter (Thermo Fisher Scientific, Schwerte, Germany). Samples were run in duplicate.

#### Foam assays

The foam-reducing abilities of the antifoam agents were tested by foaming conditioned medium (room temperature) using a magnetic stirrer at 1600 rpm. Antifoam agents were gradually added (0.0001% increments), and foam reduction was visually monitored.

#### Data analysis

Data analysis was performed in R (version 4.2.2). Linear regressions were used to estimate the 50% inhibitory concentration (IC_50_). For each antifoam agent, absorbance_570 − 690 nm_ was regressed on cell number for different antifoam concentrations. Metabolic activity was calculated for every resulting slope (using the no-antifoam control as baseline) and then regressed on concentrations; the IC_50_ was determined by the concentration corresponding to a metabolic activity of 50%. Wilcoxon signed-rank tests with continuity correction were calculated to compare the differences between two curves against zero (as these differences often do not follow a normal distribution; two-sided tests were used if not indicated otherwise).

## Results

### Metabolic changes of adherent cell culture following antifoam treatment

**Fig. 1:**
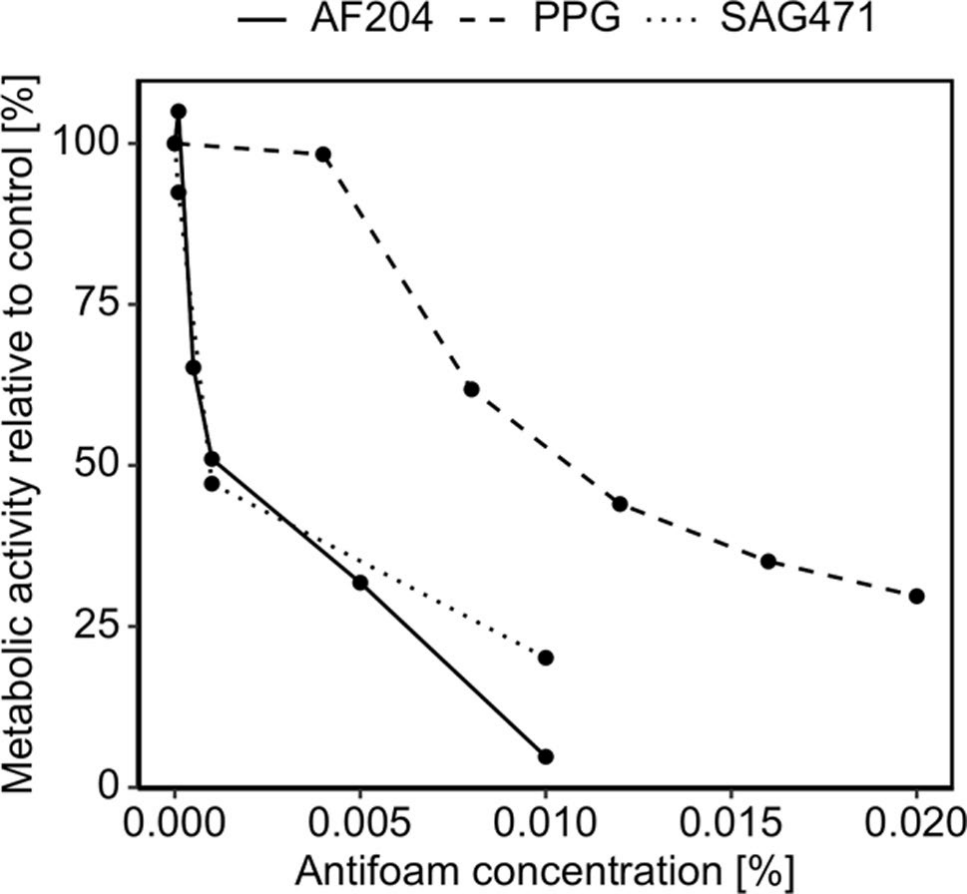
Metabolic activity of *Spodoptera frugiperda* (Sf) 9 cells in response to different concentrations of Antifoam 204 (AF204), polypropylene glycol (PPG), and a silicone antifoam compound (SAG471), relative to the no-antifoam control. Metabolic activity was estimated by regressing absorbance_570 − 690 nm_ on cell number measured in MTT assay. See supplemental Figure S3 for confidence intervals indicating statistically significant changes

After incubating Sf9 cells grown in adherent culture with different antifoam agents for 24 h, a concentration-dependent and agent-specific change in cell morphology was observed (see supplemental Figure S1). At increasing concentrations, cell walls appeared blurred, and the intracellular granularity increased compared to the no-antifoam control. For each agent and concentration, absorbance values from the MTT assay were plotted against cell number (see supplemental Figure S2). Standard lines were generated, and their slopes were used to determine the dose-response curve of Sf9 cells to stated antifoams (Fig. 1). While PPG showed only a modest inhibition of metabolic activity at concentrations up to 0.004%, AF204 and SAG471 already inhibited cell growth severely at low concentrations. Visually, AF204 increased growth at concentrations as low as 0.0001%; however, this improvement was not statistically significant as shown in supplemental Figure S3. A 50% inhibitory concentration (IC_50_) of 0.0035% for AF204, 0.0129% for PPG and 0.0043% for SAG471 was calculated. Thus, Sf9 cells tolerated approximately three times higher concentrations of PPG compared to AF204 and SAG471.

### Antifoam-induced altered growth behavior of suspension culture

**Fig. 2:**
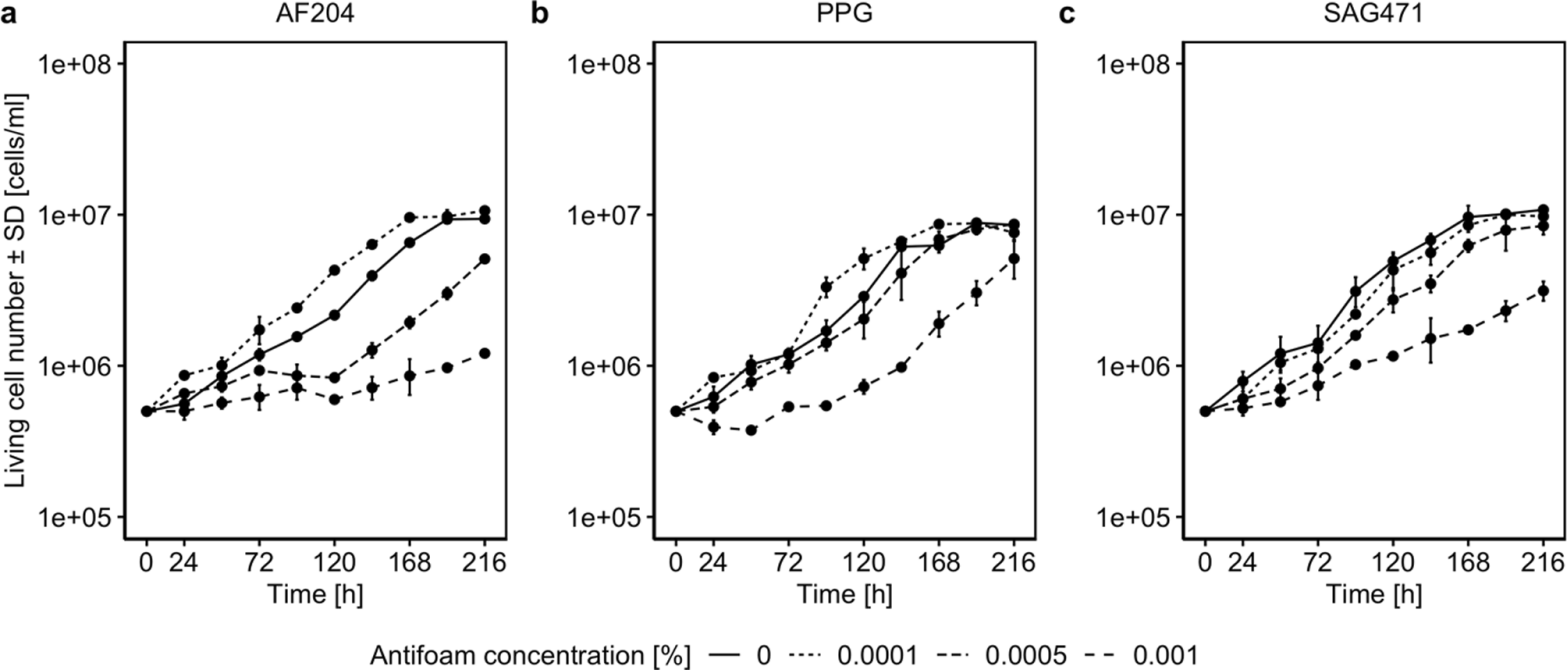
Growth curve of *Spodoptera frugiperda* (Sf) 9 cells depending on different concentrations of (**a**) Antifoam 204 (AF204), (**b**) polypropylene glycol (PPG), and (**c**) a silicone antifoam compound (SAG471); *n* = 2. Error bars indicate standard deviations

Based on the IC_50_ values calculated in the previous experiment, growth parameters of Sf9 cells in suspension were determined from growth curves in response to different antifoam agent concentrations (Fig. 2; statistical comparisons are summarized in Table 1). At a final concentration of 0.0001%, AF204 significantly enhanced cell growth by minimizing the lag phase and population doubling time (PDT). However, at higher concentrations (0.0005% and 0.001%) cell growth was significantly reduced compared to the no-antifoam control (Fig. 2a). For PPG, a final concentration of 0.0001% also promoted cell growth as indicated by a shortened lag time and an increased specific growth rate (µ), while 0.001% resulted in a significantly reduction in cell growth (Fig. 2b). In the case of SAG471, cell growth at 0.0001% and 0.0005% was comparable for the no-antifoam control in terms of PDT and µ. However, lag times increased, and living cell numbers were significantly lower at all concentrations (Fig. 2c). Across all antifoam agents, the highest concentration led to reduced living cell numbers and lower maximum cell densities. Additionally, except for SAG471, considerably prolonged lag times were observed at higher concentrations. As in the adherent culture, Sf9 cells showed increased tolerance to PPG. In contrast to AF204, SAG471 also demonstrated improved tolerance in suspension culture.

**Table 1:** Growth parameters of *Spodoptera frugiperda* (Sf) 9 cells depending on different concentrations of antifoam agents. Parameters were determined according to Murhammer [23]

Antifoam agent	Concentration [%]	Lag time [h]	Exponential phase [h]	µ [h^− 1^]	PDT [h]	Δ
AF204	0	20.10	24–192	0.01678	41.31	
	0.0001	0.66	0–168	0.01754	39.52	V = 0, *p* = 0.004
	0.0005	77.07	96–216	0.01572	44.10	V = 44, *p* = 0.008
	0.001	95.42	120–216	0.00716	96.79	V = 45, *p* = 0.004
PPG	0	11.68	24–192	0.01643	42.19	
	0.0001	3.38	0–168	0.01808	38.33	V = 11, *p* = 0.203
	0.0005	26.93	24–168	0.01724	40.21	V = 37, *p* = 0.098
	0.001	98.75	96–216	0.01911	36.27	V = 45, *p* = 0.004
SAG471	0	1.16	0–168	0.01810	38.29	
	0.0001	9.68	24–192	0.01745	39.72	V = 45, *p* = 0.004
	0.0005	29.50	48–192	0.01744	39.75	V = 45, *p* = 0.004
	0.001	31.09	48–216	0.00961	72.15	V = 45, *p* = 0.004

*Note* Lag time: time before exponential phase; µ: specific growth rate; PDT: population doubling time; Δ: result of Wilcoxon signed rank test (with continuity correction), checking if there is a difference of living cell numbers between the respective concentration and 0%; *n* = 2

The cells’ viability was similar to the no-antifoam control (see supplement Figure S4). Furthermore, in all no-antifoam controls a rim of cells and cell fragments formed during cultivation (see supplemental Figures S5 and S6), whereas this effect was reduced or absent with increasing antifoam concentrations.

### Influence of antifoam on the infectivity of baculovirus

Following the investigations of antifoam effects on cell growth, their impact on baculovirus infectivity was examined. Therefore, a plaque assay (*n* = 2) was performed after incubating three virus stock aliquots with one antifoam agent each (final concentration: 0.0001%) for 30 min. The mean virus titer for the no-antifoam control was M = 5.55 × 10^7^ pfu/ml (SD = 3.90 × 10^7^ pfu/ml; see supplemental Table S1). The titers for AF204 (M = 5.47 × 10^7^ pfu/ml, SD = 3.76 × 10^7^ pfu/ml), PPG (M = 4.29 × 10^7^ pfu/ml, SD = 1.33 × 10^7^ pfu/ml) and SAG471 (M = 4.45 × 10^7^ pfu/ml, SD = 2.17 × 10^7^ pfu/ml) were comparable.

### Altered infection dynamics caused by antifoam addition

**Fig. 3:**
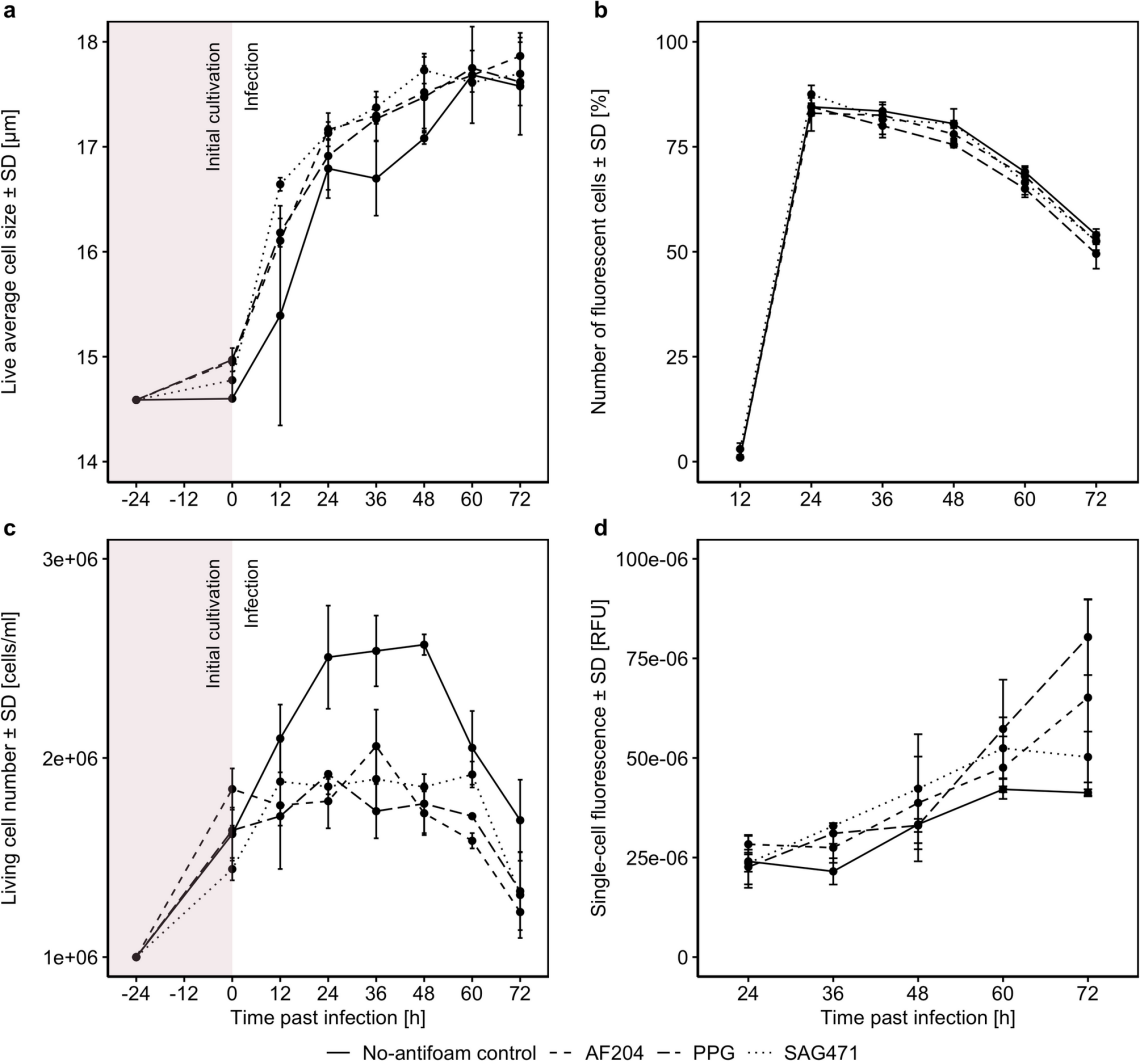
Influence of different antifoam agents (Antifoam 204, AF204; polypropylene glycol, PPG; silicone antifoam compound, SAG471; final concentration: 0.0001%) on (**a**) cell size, (**b**) share of fluorescent cells, (**c**) living cell number and (**d**) single-cell fluorescence (calculated by dividing the mean fluorescence of fluorescent cells by the number of fluorescent cells) before and after infection of *Spodoptera frugiperda* (Sf) 9 cells with baculovirus expressing mCherry (MOI = 1), *n* = 2. Error bars indicate standard deviations

24 h after start of Sf9 cultivation in suspension (and before infection), an increase in cell size was observed when antifoam agents were present (Fig. 3a). The difference sustained throughout the infection cycle, but statistical significance was only observed for PPG (V = 0, *p* = 0.031) while AF204 (V = 1, *p* = 0.063) and SAG471 (V = 1, *p* = 0.063) did not reach significance.

In the no-antifoam control the living cell number increased after infection and declined 36 h later, whereas in cultures treated with antifoam, cell numbers stagnated for 60 h after infection (Fig. 3c; test of differences to the no-antifoam control: AF204: V = 21, *p* = 0.031; PPG: V = 21, *p* = 0.031; SAG471: V = 21, *p* = 0.031).

The share of fluorescent cells was not affected by antifoam use, with a peak at 24 h past infection, followed by a gradual decline (Fig. 3b). Statistical tests did not reveal significant differences compared to the no-antifoam control (AF204: V = 15, *p* = 0.058; PPG: V = 10, *p* = 0.100; SAG471: V = 7.5, *p* = 0.999).

Based on the previous results, we assumed that the addition of antifoam agents would increase the protein production, which should be observable in an increase of single-cell fluorescence due to an increased mCherry production. Indeed, our analysis showed that fluorescence (calculated by dividing the mean fluorescence of fluorescent cells by the number of fluorescent cells) increased over time (Fig. 3d). One-sided Wilcoxon signed-rank tests revealed that fluorescence was significantly higher when AF204 was added compared to the no-antifoam control (V = 0, *p* = 0.031), but no significant differences were observed for PPG (V = 3, *p* = 0.156) and SAG471 (V = 1, *p* = 0.063).

### Differences in foam-reducing abilities

The foam-reducing efficiency of the antifoam agents was tested by incrementally adding them to foamed medium (0.0001% per step). Among the tested agents, SAG471 demonstrated the highest efficiency, substantially reducing foam at a final concentration of 0.0001% (see supplemental Figure S7). In contrast, AF204 required concentrations above 0.0016%, and PPG above 0.001%, to achieve noticeable foam reduction—concentrations that had previously been shown to inhibit cell growth.

## Discussion

### Altered growth behavior of Sf9 cells following antifoam treatment

Across two experiments, we studied the effects of the antifoam agents AF204, PPG and SAG471 on Sf9 cell growth and viability. In adherent culture, higher antifoam concentrations resulted in reduced metabolic activity of cells. However, PPG was found to have a threefold higher IC_50_ compared to AF204 and SAG471, indicating less detrimental effects. Further investigations into the reasons for these differences could be useful but would require insight into the antifoams’ composition.

In suspension cultures, higher antifoam concentrations also led to reduced cell growth, including for PPG, despite its higher IC_50_. Similar trends in viability suggest an antifoam-induced cell cycle arrest as a possible cause. However, at 0.0001% both AF204 and PPG improved cell growth compared to the no-antifoam control. This may be attributed to a protective effect against hydrodynamic and mechanical damage, similar to Pluronic F-68 [14]. This assumption is supported by reduced rim formations, consisting of cells and cell debris, in the presence of antifoam agents. However, increasing the concentration of AF204 causes much severe effects on cells than SAG471 and PPG. The aversive effect of high PPG concentrations in suspension contrasts with the high IC_50_ in adherent culture. Similarly, while metabolic activity in adherent culture was found to be comparable for AF204 and SAG471, their effects differed in suspension. This may be explained by culture form dependent alterations in cell physiology and metabolism described in previous research [24, 25].

Although direct comparisons with previous studies are challenging due to differences in organisms and antifoam agents used, our data suggests that Sf9 cells tolerate notably lower concentrations. In *E. coli* K-12 silicone-based antifoam had no negative effects at concentrations up to 0.025% [7], while in *S. cerevisiae* cultures remained unaffected at concentrations below 8% [26]. In *P. pastoris*, a vegetable-based alkoxylated fatty acid ester supported steady growth up to 8%, whereas silicone-based antifoams reduced specific growth rate at just 0.8% [10, 26]. In CHO cells, growth remained unchanged with certain antifoams up to 0.003%, while others (including AF204) were toxic at the same concentration [11]. The fact that CHO cell growth was either unaffected or completely inhibited by antifoam agents of similar composition highlights the challenge of generalizing results across different cell systems. Therefore, each antifoam agent must be evaluated individually, including for Sf9 cell culture.

### Altered infection dynamics in the presence of antifoam

Since Sf9 cells are commonly used in the baculovirus expression system, we examined the effects of antifoam agents on baculovirus infectivity and infection dynamics. Virus titers determined after an initial 30 min incubation of baculovirus with antifoam were comparable between all conditions, suggesting that viral infectivity is not reduced by short-term exposure. Infection assays further indicated that the addition of antifoam did not affect the share of fluorescent cells. However, the agents led to a notable difference in cell growth. In the no-antifoam control, the cell number increased after infection while it stagnated when antifoams were present, suggesting that the infection is more effective. This difference may be attributed to changes in plasma membrane characteristics that enhance virus attachment and internalization [12]. Furthermore, cells exposed to antifoam showed slightly larger sizes and lysed approximately 24 h later than cells in the no-antifoam control, potentially prolonging protein production and benefitting recombinant protein yields. Indeed, the latter assumption was supported by stronger single-cell fluorescence in the presence of all antifoam agents tested, indicating a trend towards higher mCherry production. This effect was significant for AF204 but not for PPG and SAG471. Therefore, it may be worthwhile to further test these agents for their influence on protein productivity in virus-based expression systems.

## Conclusion

Overall, our findings indicate that antifoam agents can have both beneficial and inhibitory effects on Sf9 cell growth, depending on their composition and concentration. A reduction in metabolic activity can be prevented by maintaining low concentrations, which even enhanced living cell numbers for AF204 and PPG. It is important to note that the insect cell culture medium used in this study already contained Pluronic F-68. Consequently, the effects of antifoam agents may differ in media lacking such additives.

Additionally, optimal antifoam concentrations may differ in large-scale bioreactors, where additional aeration and agitation can influence foam formation and cell behavior. This is particularly relevant considering the lack of defoaming effects of AF204 and PPG within their non-toxic concentration range. Among the tested agents, SAG41 was found to be the most promising candidate, due to its low impact on cell growth and effective foam-reducing properties. However future validation in industry-scale applications is necessary to confirm these findings.

Besides cell growth, adjusting antifoam concentrations may also improve baculovirus infection dynamics, potentially leading to increased recombinant protein yields. While most virus-based expression systems in higher cells prioritize antifoam use for foam control, our results suggest that antifoam agents could also improve infection and thus increase production efficiency in those systems. Finally, the assumption of an antifoam-dependent change in cell membrane properties presents the possibility of using these agents to enhance transfection efficiency of exogenic material, broadening their applications beyond foam control.

## Electronic supplementary material

Below is the link to the electronic supplementary material.

Supplementary Material 1

## Data Availability

The datasets generated during the current study and the analysis script are available in the Open Science Framework (OSF), 10.17605/OSF.IO/XM79T.

## Abbreviations

AF204: Antifoam 204
CHO: Chinese hamster ovary cells
µ: Specific growth rate
M: Mean virus titer
MTT: 3-(4,5-dimethylthiazol-2-yl)2,5-diphenyltetrazolium bromide
n: Number of biological replicates
PDT: Population doubling time
PPG: Polypropylene glycol
RFU: Relative fluorescence unit
SAG471: Silicone antifoam compound
SD: Standard deviation
