# Supplementary material for: Effects of antifoam agents on Spodoptera frugiperda 9 cell growth and baculovirus infection dynamics
Source: J Biol Eng. 2025 May 10;19:43. doi: 10.1186/s13036-025-00516-w (PMC12065184; doi:10.1186/s13036-025-00516-w)
Supplement: Supplementary file 1 — Supplementary Material 1 [file 13036_2025_516_MOESM1_ESM.docx]

**Additional file 1: Effects of antifoam agents on *Spodoptera frugiperda* 9 cell growth and baculovirus infection dynamics**

Kristina Worch^1*^, Merlin Krause^1^, Antje Burse^1^

^1^ Department of Medical Engineering and Biotechnology, Ernst-Abbe-Hochschule, University of Applied Sciences, Jena, Germany

* Correspondence to:

Kristina Worch, [kristina.worch@eah-jena.de](mailto:kristina.worch@eah-jena.de)

|  | AF204 | PPG | SAG471 |
| --- | --- | --- | --- |
| 0 % | 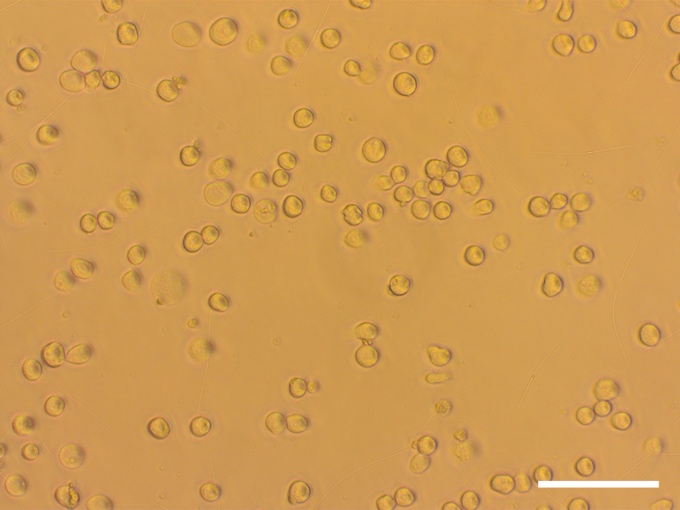 | 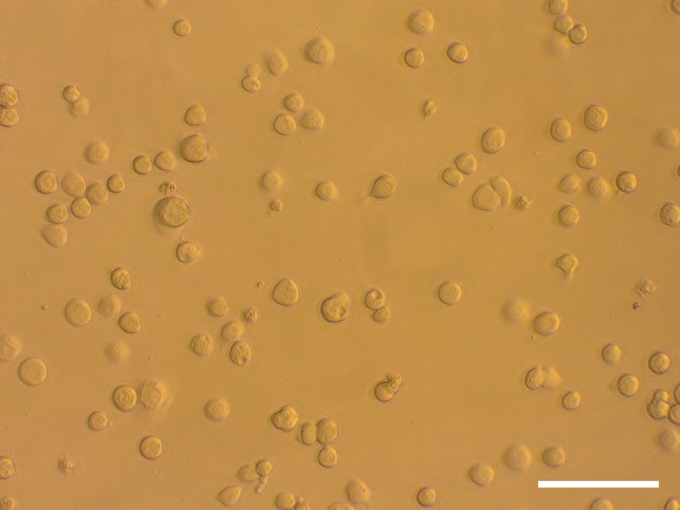 | 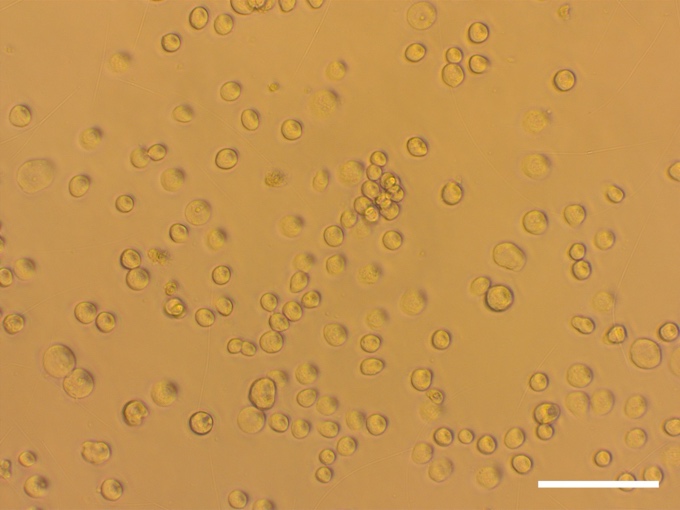 |
| 0.0001 % | 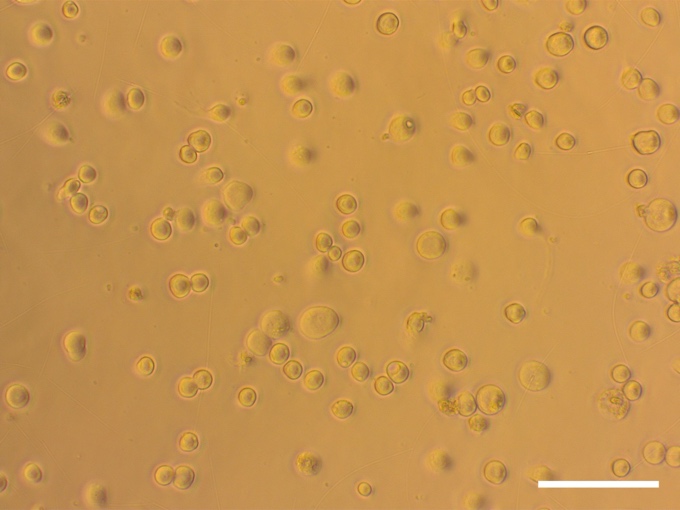 | 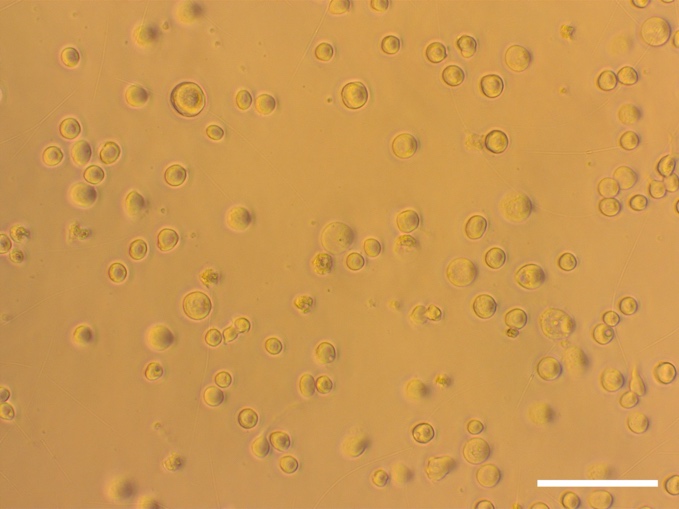 | 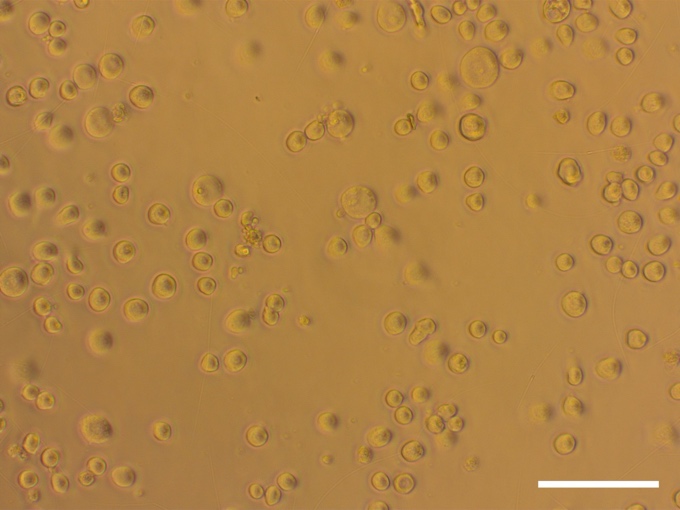 |
| 0.001 % | 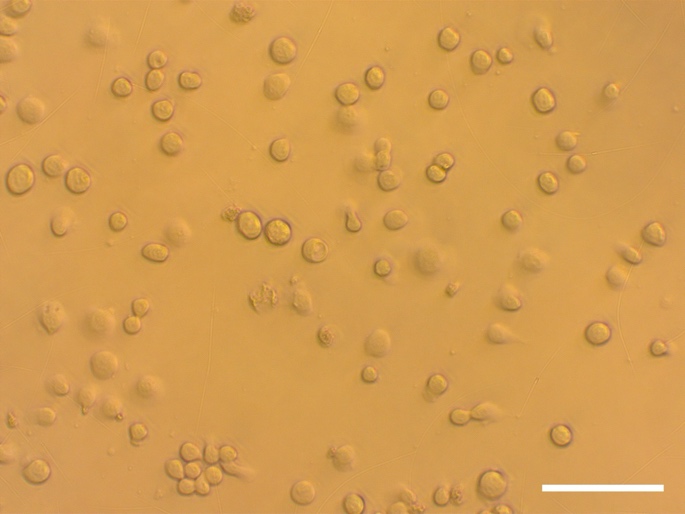 | 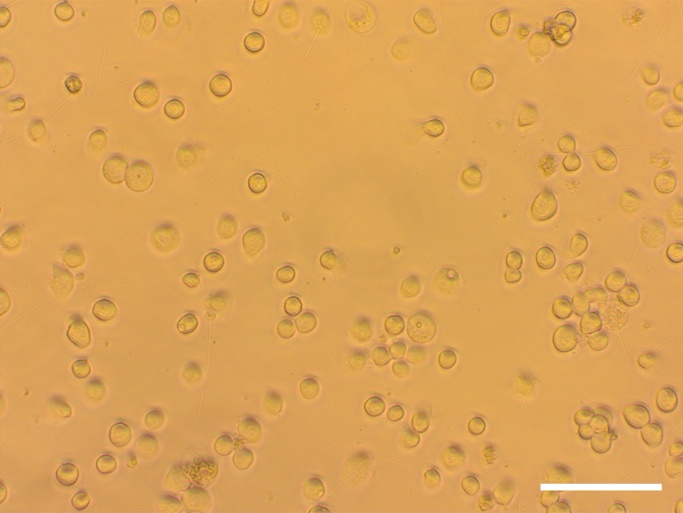 | 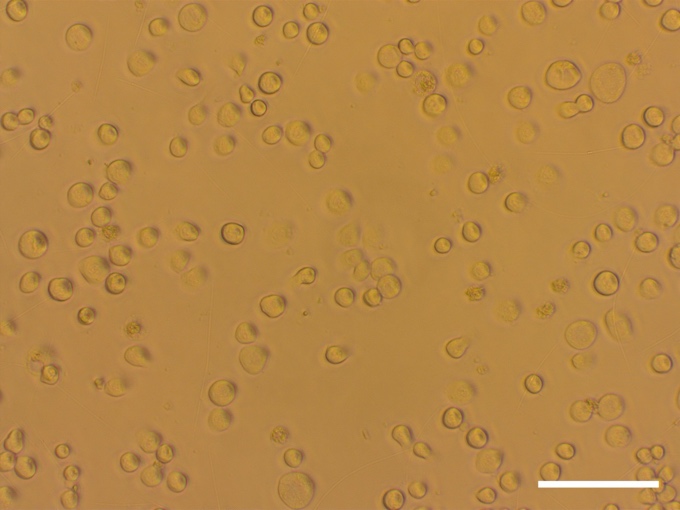 |
| 0.01 % | 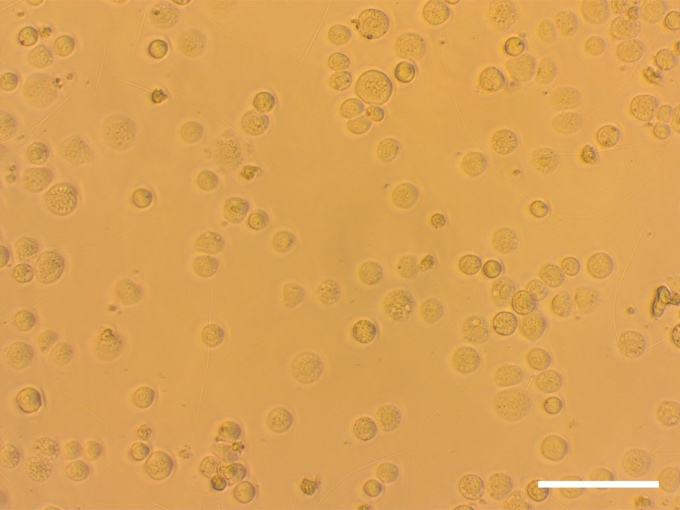 | 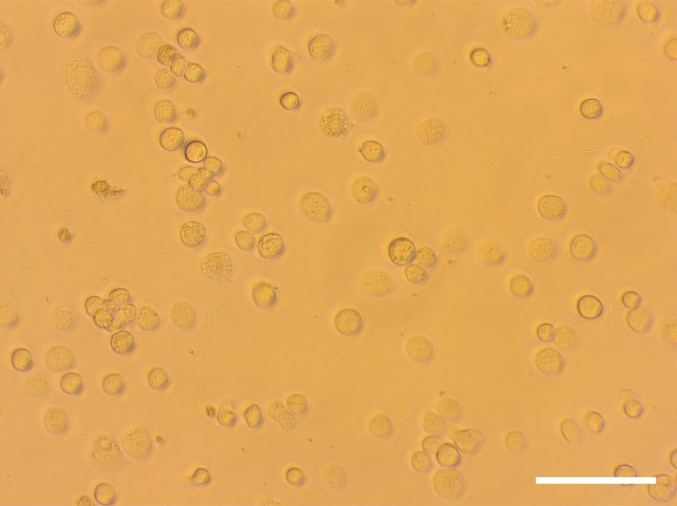 | 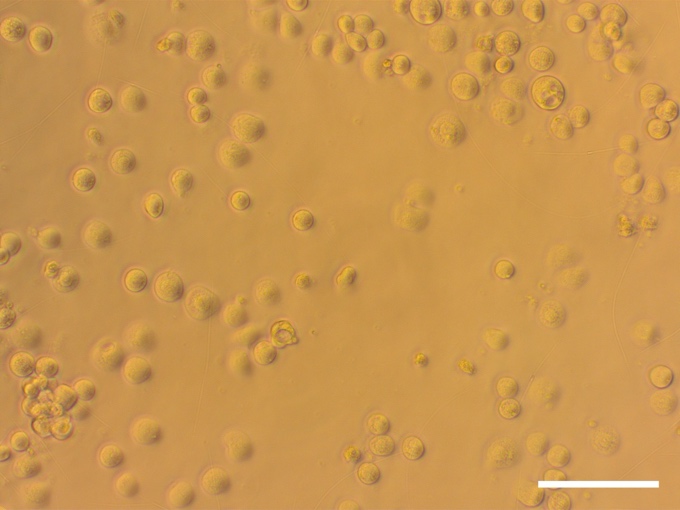 |

**Figure S1** Morphological changes of *Spodoptera frugiperda* (Sf) 9 cells in response to different concentrations of antifoam agents: Antifoam 204 (AF204), polypropylene glycol (PPG), silicone antifoam compound (SAG471); scale bars: 100 µm.


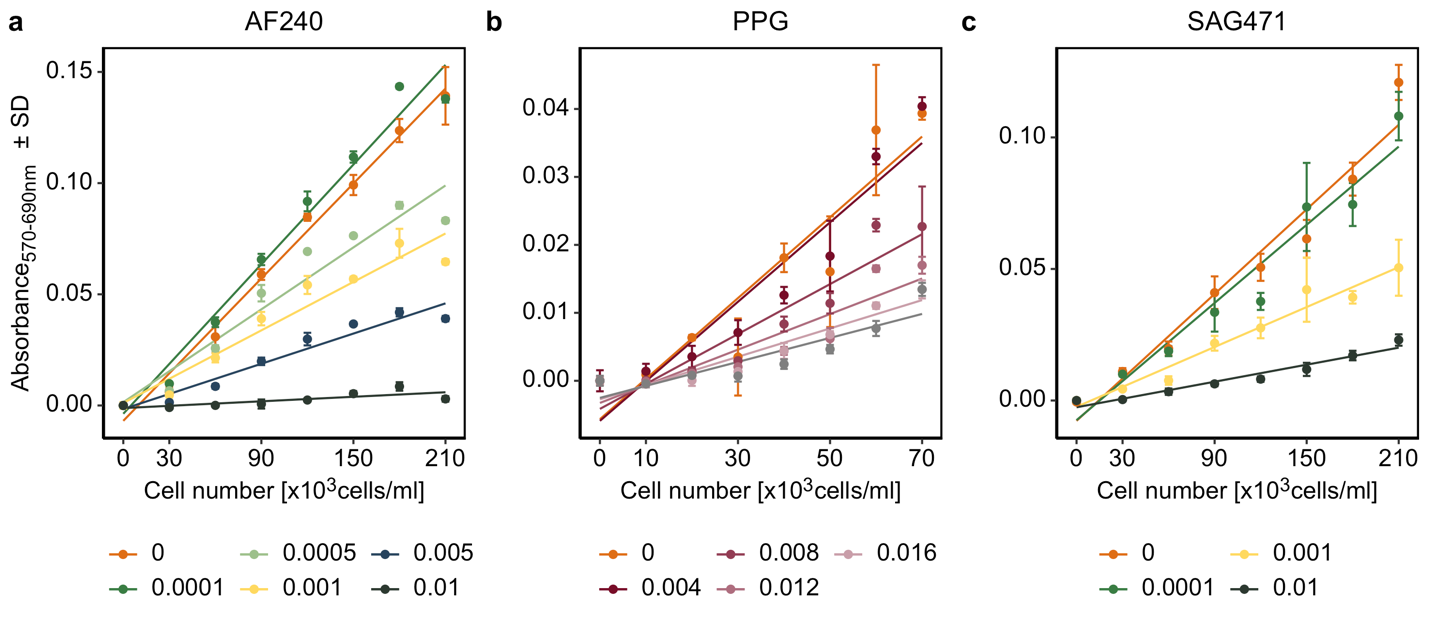


**Figure S2** Correlation between absorbance_570-690 nm_ and cell count of *Spodoptera frugiperda* (Sf) 9 cells following MTT assay after 24 h incubation with different concentrations of antifoam agents: (a) Antifoam 204 (AF204), (b) polypropylene glycol (PPG), (c) silicone antifoam compound (SAG471); n = 2. Error bars indicate standard deviations.


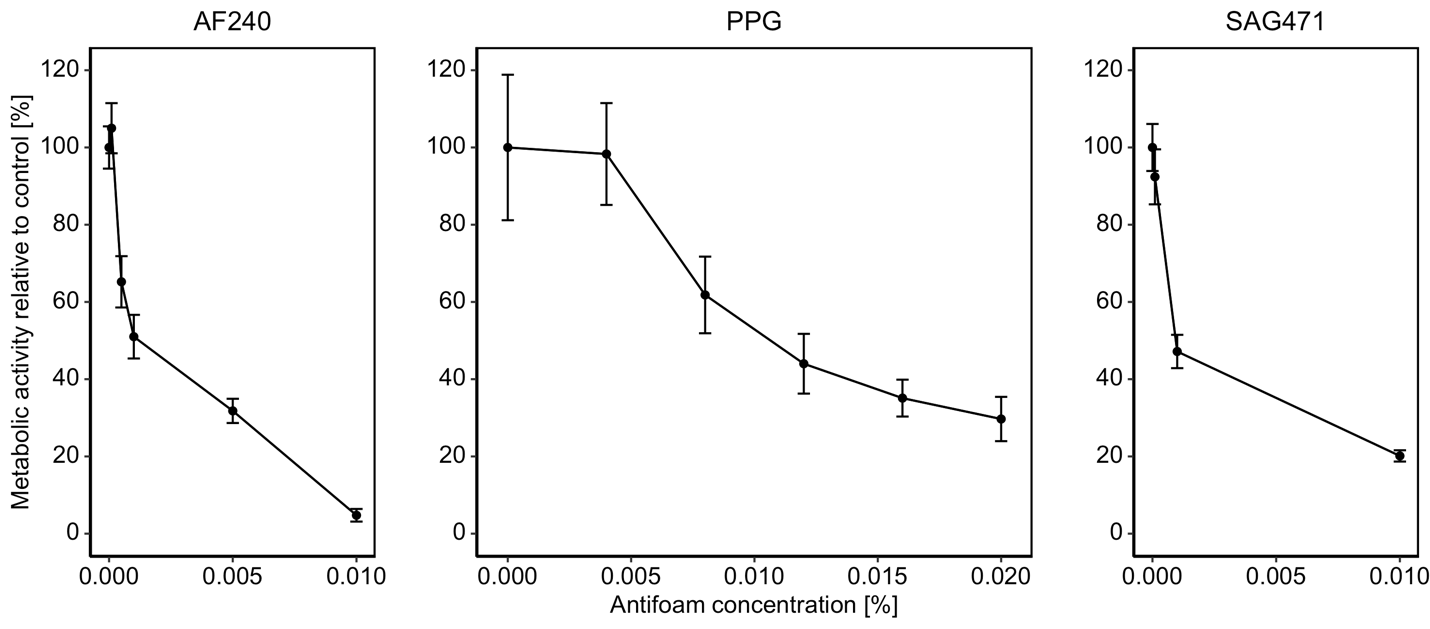


**Figure S3** Metabolic activity of *Spodoptera frugiperda* (Sf) 9 cells in response to different concentrations of Antifoam 204 (AF204), polypropylene glycol (PPG), and a silicone antifoam compound (SAG471), relative to no-antifoam control. Metabolic activity was estimated by regressing absorbance_570-690 nm_ on cell number measured in MTT assay, error bars visualize 95 % confidence intervals, indicating significant changes.


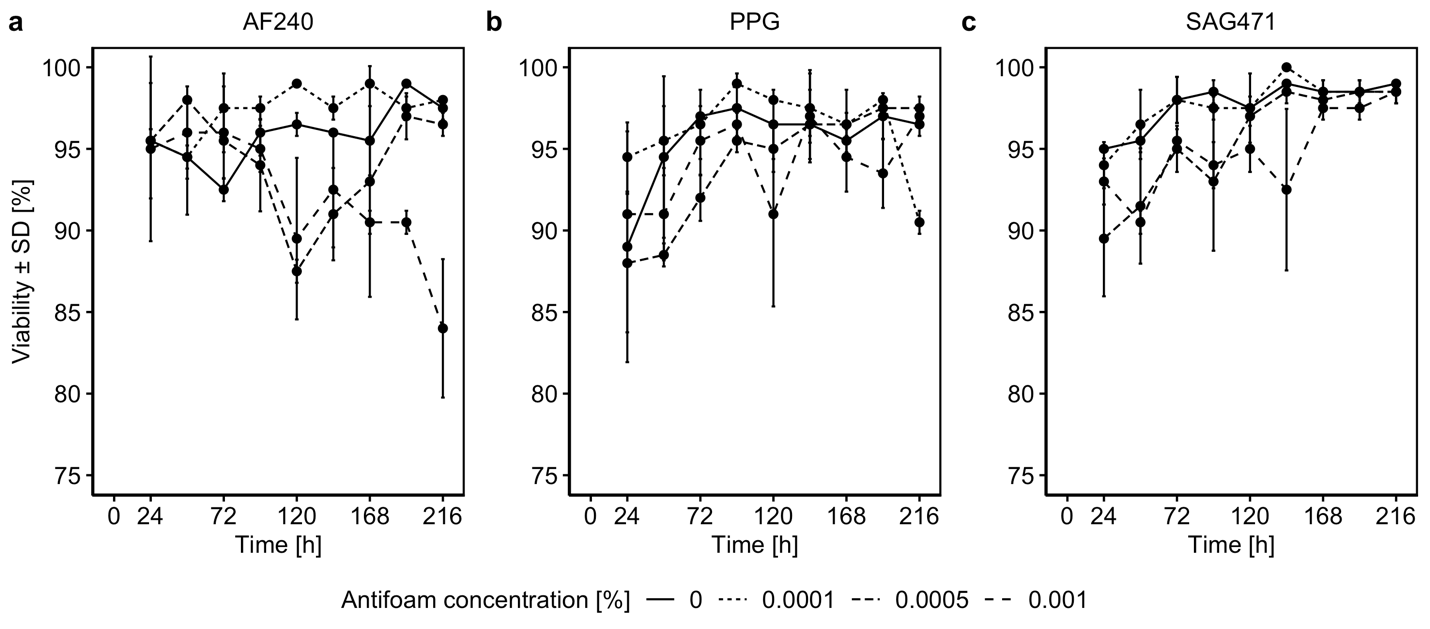


**Figure S4** Viability of *Spodoptera frugiperda* (Sf) 9 cells depending on different concentrations of antifoam agents (a) Antifoam 204 (AF204), (b) polypropylene glycol (PPG), and (c) a silicone antifoam compound (SAG471); n = 2. Error bars indicate standard deviations.

|  | AF204 | PPG | SAG471 |
| --- | --- | --- | --- |
| 0 % | 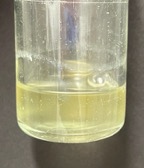 | 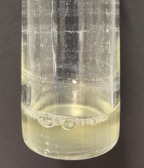 | 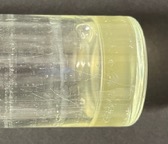 |
| 0.0001 % | 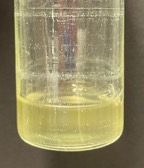 | 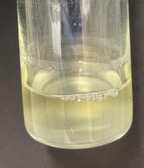 | 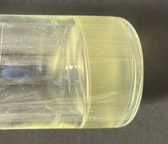 |
| 0.0005 % | 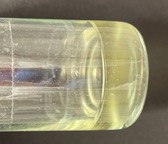 | 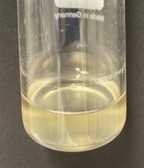 | 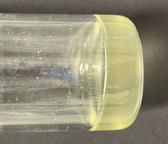 |
| 0.001 % | 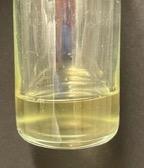 | 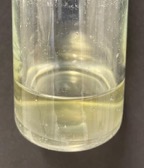 | 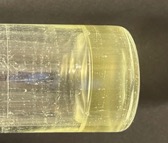 |

**Figure S5** Rim formation on day 9 of cultivation of Spodoptera frugiperda (sf) 9 cells in silanized 50 ml culture flasks in Insect-XPRESS™ medium supplemented with different antifoam agents: Antifoam 204 (AF204), polypropylene glycol (PPG), silicone antifoam compound (SAG471).


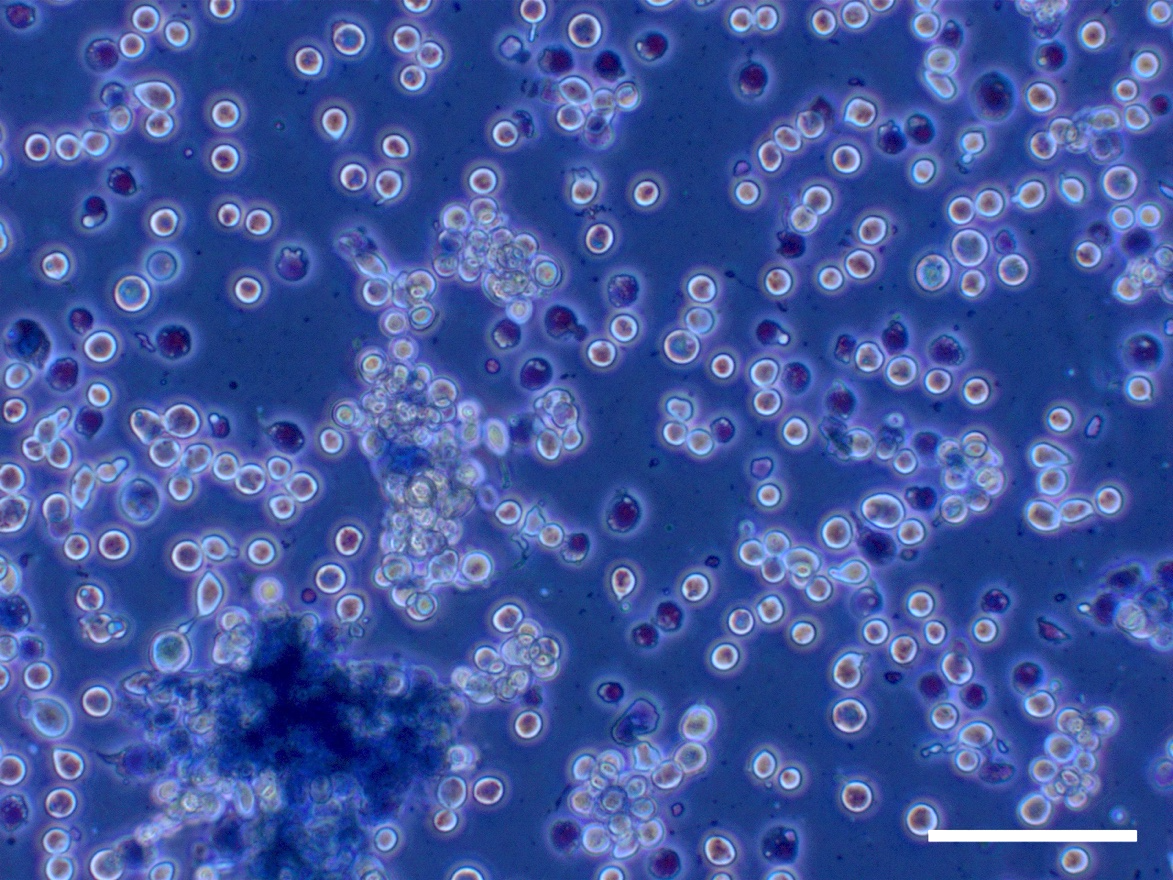


**Figure S6** Composition of rim formation visualized by residue homogenization in Insect-XPRESS™ medium and trypan blue staining; scale bar: 100 µm.

| **a** | **b** | **c** |
| --- | --- | --- |
| **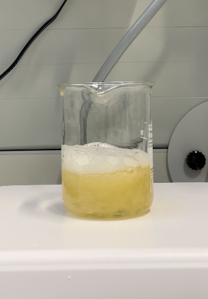** | **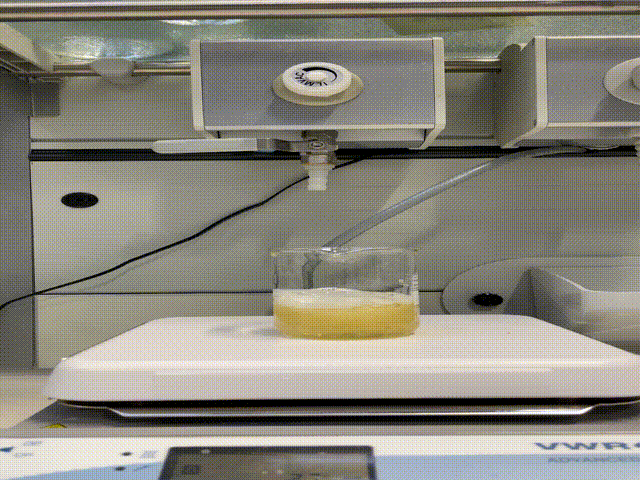** | **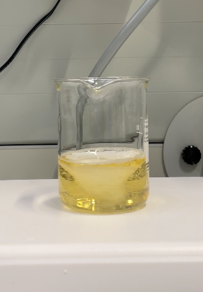** |

**Figure S7** Visual control of influence of SAG471 (0.0001 %) on foam formation in conditioned Insect-XPRESS™ medium: (a) medium before addition of SAG471, (b) foam reduction by adding SAG471, (c) medium approximately 1 min after addition of SAG471.

**Table S1** Number of plaques (P) determined by plaque assay. Virus was incubated with Antifoam 204 (AF204), polypropylene glycol (PPG), and a silicone antifoam compound (SAG471) before application to wells (final concentration: 0.0001 %).

| **Virus dilution** | **No antifoam control** | | **AF204** | | **PPG** | | **SAG471** | |
| --- | --- | --- | --- | --- | --- | --- | --- | --- |
|  | **P** | **Titer [x 10^7^ pfu/ml]** | **P** | **Titer [x 10^7^ pfu/ml]** | **P** | **Titer [x 10^7^ pfu/ml]** | **P** | **Titer [x 10^7^ pfu/ml]** |
| PC |  |  |  |  |  |  |  |  |
| PC |  |  |  |  |  |  |  |  |
| 10^-5^ | 185 | 2.31 |  |  |  |  | 171 | 2.14 |
| 10^-5^ | 161 | 2.01 | 105 | 1.31 | 175 | 2.19 | 177 | 2.21 |
| 10^-6^ | 40 | 5.00 | 45 | 5.63 | 43 | 5.38 | 50 | 6.25 |
| 10^-6^ | 46 | 5.75 | 37 | 4.63 | 41 | 5.13 | 39 | 4.88 |
| 10^-7^ | 7 | 8.75 | 3 | 3.75 | 4 | 5.00 | 6 | 7.50 |
| 10^-7^ | 2 | 2.50 | 4 | 5.00 | 3 | 3.75 | 3 | 3.75 |
| 10^-8^ |  |  |  |  |  |  |  |  |
| 10^-8^ | 1 | 12.5 | 1 | 12.5 |  |  |  |  |
| NC |  |  |  |  |  |  |  |  |
| NC |  |  |  |  |  |  |  |  |
| **Mean** |  | **5.55** |  | **5.47** |  | **4.29** |  | **4.45** |
| **SD** |  | **3.90** |  | **3.76** |  | **1.33** |  | **2.17** |

Note: PC: positive control with dilution of 10^-3^; NC: negative control without virus; SD: standard deviation; n = 2.
